# Immunoinformatics-driven design of a multi-epitope vaccine against *Clostridium perfringens* in yaks

**DOI:** 10.3389/fimmu.2026.1857374

**Published:** 2026-06-29

**Authors:** Dan Wu, Runbo Luo, Kexin Li, Yulin Peng, Xiao Yue, Yurui Wang, Haoyu Fan, Yifang Wen, Yixin Huang, Sizhu Suolang, Suizhong Cao

**Affiliations:** 1 College of Veterinary Medicine, Sichuan Agricultural University, Chengdu, Sichuan, China; 2 Key Laboratory for Prevention and Control of Hydatid Disease in Xizang (Co-constructed by Ministry and Province), Ministry of Agriculture and Rural Affairs, College of Animal Science, Xizang Agricultural and Animal Husbandry University, Linzhi, Xizang, China

**Keywords:** *Clostridium perfringens*, immunoinformatics, molecular docking, multi-epitope vaccine, yak

## Abstract

**Introduction:**

*Clostridium perfringens* is the primary causative agent of enterotoxemia in yaks, resulting in substantial economic losses on the Qinghai-Tibet Plateau. Conventional vaccines exhibit limited protective breadth and suboptimal efficacy, highlighting the need for innovative strategies. Here, we aimed to construct a novel vaccine candidate incorporating epitopes from multiple prevalent toxinotypes (A, C, E) of *C. perfringens* affecting yaks, using immunoinformatics approach.

**Methods:**

A hierarchical immunoinformatics pipeline was implemented, encompassing subtractive genomics to identify core virulence factors, prediction and filtering of immunogenic T-cell and B-cell epitopes, rational multi-epitope vaccine design incorporating adjuvant and linkers, three-dimensional structure modeling and validation, molecular docking to evaluate interactions with TLR4, molecular dynamics simulations to confirm complex stability, and codon optimization to facilitate heterologous expression.

**Results and discussion:**

Five core virulence proteins (*Iap*, *CpsE*, *NanH*, *Plc*, *Pfo*) were identified from genomic data, leading to the prediction and selection of ten cytotoxic T lymphocyte (CTL) epitopes, five helper T lymphocyte (HTL) epitopes, and five B-cell epitopes. The final 352-amino-acid multi-epitope vaccine (MEV) construct was assembled using the adjuvant human β-defensin-3 and specific linkers (AAY, GPGPG, KK). Computational evaluations confirmed the vaccine’s high antigenicity (VaxiJen score: 0.9092), non-allergenic nature, and structural stability. Molecular docking revealed strong binding affinity with TLR2 (-1024.6 kcal/mol) and TLR4 (-1104.4 kcal/mol). Molecular dynamics simulations over 100 ns confirmed stable TLR4 complex with an average RMSD of 0.1971 ± 0.0377 nm, while the TLR2 complex showed an average RMSD of 0.2692 ± 0.0420 nm. Immune simulation profiles predicted the induction of robust humoral and cellular immune responses, including elevated antibody titers, T-cell activation, and cytokine production. *In silico* cloning verified the potential for efficient expression in E. coli.

**Conclusion:**

This study designed a novel multi-epitope vaccine against *C. perfringens* in yaks using an immunoinformatics approach. The vaccine showed high antigenicity, stability, and broad allelic coverage *in silico*, providing a promising candidate that requires rigorous *in vitro* and *in vivo* experimental validation to confirm these computational predictions. This work offers a foundation for the development of effective vaccines against yak *C. perfringens* infections on the Qinghai-Tibet Plateau.

## 1 Introduction

The yak (*Bos grunniens*) is a cornerstone of livelihood and cultural heritage for communities on the Qinghai-Tibet Plateau. As a primary source of meat, milk, fiber, and draft power, yaks are crucial for the region’s economy and food security (1). However, infectious diseases pose a major threat to yak health and productivity. Among them, enterotoxemia, a frequently fatal gastrointestinal disease, is a key concern (2). This disease is primarily caused by *C. perfringens*, a gram-positive, spore-forming, anaerobic bacterium (3).

In traditional typing methods, *C. perfringens* is classified into five toxinotypes (A–E) based on the production of four major lethal toxins (alpha, beta, epsilon, iota) (4). While *C. perfringens* affects various livestock, enterotoxemia in yaks presents unique epidemiological challenges. In contrast to lowland cattle, sheep, or pigs, where prevalence ranges from 45% to 70% and intensive farming amplifies transmission risks, yak infections are shaped by high-altitude, low-temperature, and strong UV conditions. Our previous study demonstrated that toxigenic type A, C, and E predominate in yaks, with infection rates ranging between 19.35% and 37.40% (2). Critically, yaks face co-circulation of toxinotypes A, C, and E, whereas most other livestock are dominated by a single type. Additionally, conventional vaccines show variable efficacy in yaks due to species-specific immune differences, and remote high-altitude herding limits vaccination coverage, exacerbating disease burden and economic losses on the Qinghai-Tibet Plateau (5, 6).

Currently available commercial *C. perfringens* vaccines are mostly traditional inactivated toxoid vaccines, which have limited cross-protection against multiple toxinotypes, require frequent booster immunizations, and show variable efficacy in yak herds due to species-specific immune response differences (7). No dedicated multi-epitope vaccine has been developed for yak *C. perfringens* infections on the Qinghai-Tibet Plateau, creating an urgent need for a targeted, broad-spectrum vaccine strategy.

Multi-epitope vaccines (MEVs) represent a cutting-edge strategy to address these challenges. A multi-epitope vaccine is a genetically engineered recombinant protein containing carefully selected antigenic epitopes, short peptides recognizable by the host immune system. By integrating multiple T- and B-cell epitopes from different antigens or even different strains, a single multi-epitope vaccine can theoretically elicit a comprehensive and robust immune response against multiple targets simultaneously (8). This strategy offers several advantages. It eliminates the need to use whole pathogens or toxins, thereby improving safety; focuses the immune response on protective epitopes, enhancing specificity and potency; and allows for the design of a single vaccine entity with the potential to provide cross-protection against multiple *C. perfringens* toxinotypes (9).

The field of immunoinformatics provides powerful computational tools for the rational design of such multi-epitope vaccines. This discipline applies bioinformatics algorithms to predict immunogenic epitopes, model protein structures, and simulate immune interactions, all performed *in silico*. This significantly reduces the time and cost associated with traditional vaccine development by prioritizing the most promising candidates before moving into expensive laboratory and animal trials (10).

Building upon our previous work involving the isolation and whole-genome sequencing of type A, C and E *C. perfringens* strains from yak feces, this study fully utilized the complete proteome data of these strains (2). Through a hierarchical immunoinformatics pipeline—including screening for core virulence factors, predicting and selecting immunogenic T- and B-cell epitopes, constructing, modeling, and validating the multi-epitope vaccine, assessing its interaction with the immune receptor TLR4 through docking and dynamics simulations—we aimed to develop the candidate construct. Codon optimization was performed to ensure its efficient expression, resulting in a broad-spectrum multi-epitope vaccine targeting the three toxinotypes. This work lays a solid foundation for the subsequent development and application of novel vaccines to prevent and control *C. perfringens* disease in yaks.

## 2 Materials and methods

### 2.1 Research process

**Figure 1:**
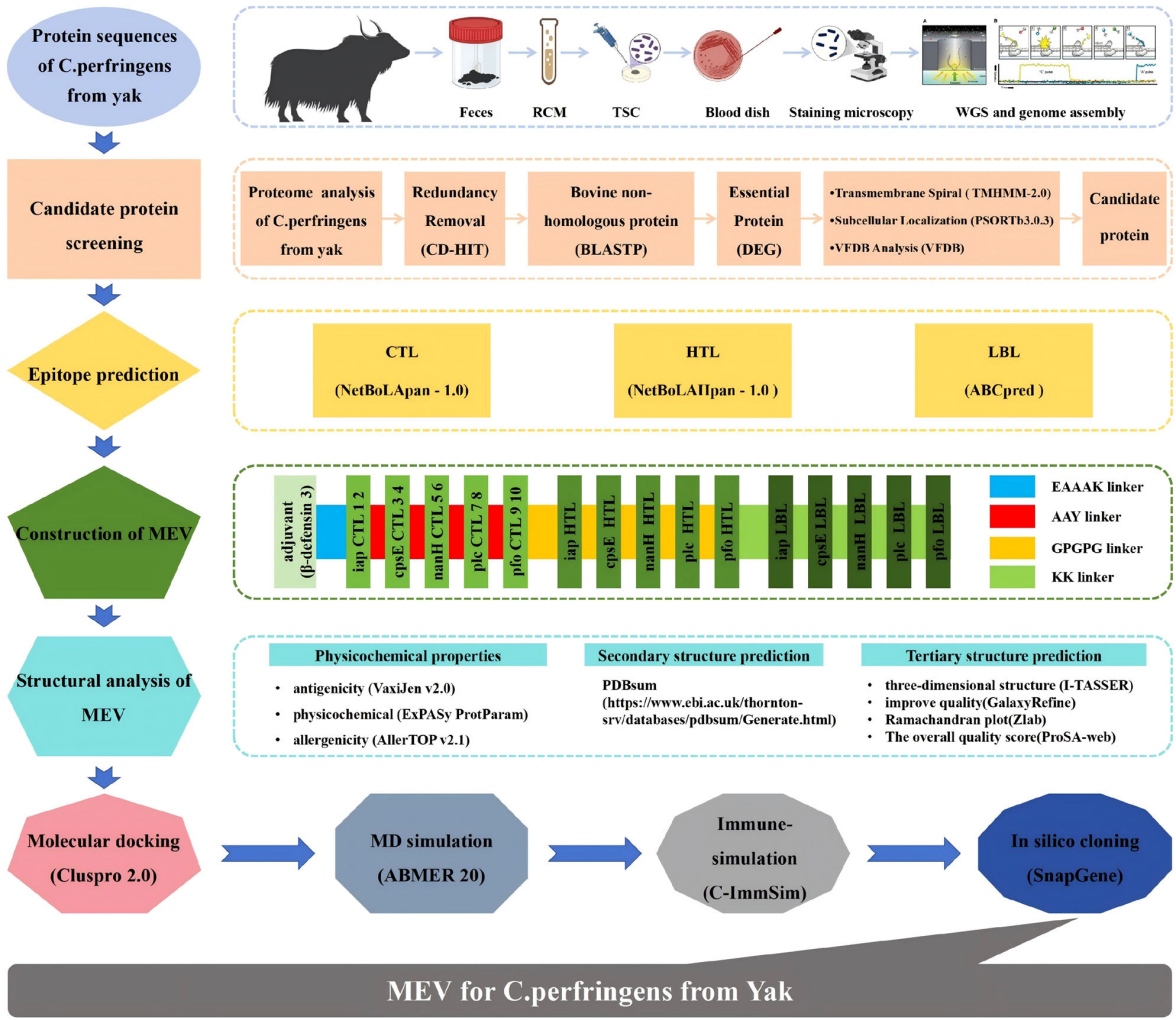
Schematic diagram of methods applied for designing an MEV against *C. perfringens* from yak.

The overall flow diagram followed for the *in silico* design of an MEV against *C. perfringens* from yak is presented in **Figure 1**.

### 2.2 Bacterial strains and protein sequence acquisition

This study originated from the *C. perfringens* strains isolated and identified from yaks in our laboratory’s previous research. Briefly, fresh yak fecal samples were collected from a pasture on the Qinghai-Tibet Plateau. After anaerobic cultivation and purification, toxinotyping was performed via multiplex PCR. Ultimately, pure cultures representing the three toxinotypes were obtained and preserved. Subsequently, library construction and whole-genome sequencing were performed by Beijing Biomarker Technologies Corporation Ltd., strictly following the standard PacBio protocols. Genome assembly was conducted using Hifiasm, and second-generation sequencing data were used for error correction with Pilon. Based on this genomic analysis, the proteomes of three representative toxigenic strains were selected to form the pathogen proteome dataset for MEV design, which was used for subsequent screening of candidate vaccine proteins. The strains used were: Type A: XZ-A1 (GenBank: CP183465), Type C: XZ-C1 (GenBank: CP183469), Type E: XZ-E1 (GenBank: CP181174). Protein sequences are provided in the **Supplementary Table**.

### 2.3 Protein sequence redundancy removal

The CD-HIT online tool (https://www.bioinformatics.org/cd-hit/) was employed to eliminate redundant protein sequences from the bacterial proteomes for subsequent analysis (11).

### 2.4 Homologous protein screening​

The full non-redundant protein sequences were subjected to BLASTP alignment​ with an E-value threshold of 1e-5 (12). Homologous proteins meeting this criterion were selected for subsequent analysis.

### 2.5 Non-homologous protein screening​

Identified homologous proteins were aligned against the bovine proteome using BLASTP (E-value threshold: 1e-5) (12). Proteins exhibiting no significant similarity to the bovine host were retained as non-homologous candidates for further analysis.

### 2.6 Essential protein screening​

Non-homologous proteins were analyzed against the Database of Essential Genes (DEG)(http://origin.tubic.org/deg/public/index.php) via BLASTP (E-value threshold: 1e-100). Proteins achieving a minimum bit score of 100 were retained as essential for pathogen viability (13).

### 2.7 Transmembrane spiral analysis

Transmembrane helices were predicted using TMHMM-2.0 (https://services.healthtech.dtu.dk/service.php?TMHMM2.0), and proteins containing more than one transmembrane helix were excluded (14). Proteins with 0 or 1 transmembrane helix are easy to clone and express; therefore, they were selected for further analysis (15).

### 2.8 Subcellular localization prediction of screened proteins​

PSORTb3.0.3 (https://www.psort.org/psortb/) was used to predict subcellular localization (16). Proteins localized to the extracellular, cell wall, or cytoplasmic membrane were selected for downstream evaluation, while all strictly intracellular proteins (including DnaA, dnaN, and other cytoplasmic proteins) were strictly excluded from further analysis.

### 2.9 VFDB analysis

To examine the virulence of surface localization proteins, all surface localization proteins were subjected to a virulence factor database (VFDB) (http://www.mgc.ac.cn/VFs/) analysis. Homologs of VFDB labeled with a bit score of >100 and an identity of >30% for proteins of *C. perfringens* were regarded as virulent (17).

### 2.10 CTL and HTL epitope prediction

For CTL and HTL epitope prediction, NetBoLApan 1.0 and NetBoLAIIpan 1.0 were used with default parameters. Epitopes were prioritised according to: (1) predicted binding rank ≤1%; (2) antigenicity score >0.5 (VaxiJen); (3) non-toxicity (ToxinPred); (4) non-allergenicity (AllerTop); and (5) ability to bind multiple BoLA alleles. Only epitopes satisfying all criteria were retained for vaccine construction. Due to >90% sequence identity between yak MHC and bovine BoLA alleles, and the lack of a dedicated yak MHC prediction server, all available bovine BoLA alleles were selected as a valid proxy for epitope prediction in this study.

For the prediction of 8, 9, 10, 11, and 12 amino acid (aa) epitopes from *C. perfringens* proteins targeting bovine MHC I (BoLA) molecules, NetBoLApan 1.0 (https://services.healthtech.dtu.dk/services/NetBoLApan-1.0/) was employed (18). Protein sequences were submitted in FASTA format. The consensus approach was selected for identification, and all the available alleles were selected for epitope prediction. The VaxiJen 2.0 server was used to evaluate antigenicity and the ability of epitopes to induce an immune response (19). Allergenicity was predicted using the AllerTop 2.0 web server (20). The ToxinPred server was utilized to identify nontoxic CTL epitopes. The top2 short peptides were selected as candidate CTL epitopes based on a comprehensive ranking for MHC I.

For bovine MHC II BoLA-DRB3 molecules, epitopes of 15 aa in length were predicted using NetBoLAIIpan 1.0 (https://services.healthtech.dtu.dk/services/NetBoLAIIpan-1.0/). Protein sequences were submitted in FASTA format, and all available alleles were selected for epitope prediction. A threshold of rank ≤ 1% was applied across all software tools. Based on the prediction results, epitope breadth (defined as the number of HLA alleles each predicted epitope could bind to) was calculated to identify epitopes capable of binding to multiple alleles. The top1 short peptides based on comprehensive ranking were selected as candidate HTL epitopes.

### 2.11 B-cell epitope prediction​

Prediction of B-cell epitopes was performed using the B-cell epitope prediction tools available in the ABCpred analysis resource (https://webs.iiitd.edu.in/raghava/abcpred/ABC_submission.html) (21). For ABCpred prediction results, the score was used as the selection criterion. Furthermore, the predicted B-cell epitopes were screened using ToxinPred, AllergenFP v.1.0, and VaxiJen to evaluate their toxicity, allergenicity, and antigenicity, respectively. The highest-scoring epitope from each protein was selected.

### 2.12 Construction of MEV

The multi-epitope vaccine was constructed using all selected epitopes. The adjuvant human β-defensin-3 was selected because it serves as a potent TLR4 agonist that strongly activates innate immunity in yaks and cattle, promoting antigen-presenting cell maturation and enhancing subsequent adaptive immune responses. The EAAAK linker was used to fuse the adjuvant with the epitope cassette, providing a rigid α-helical spacer to avoid structural interference and maintain independent folding of the adjuvant and epitopes. All B-cell, HTL, and CTL epitopes were connected via KK, GPGPG, and AAY linkers, respectively (22) (**Figure 1**).The KK linker maintains B-cell epitope integrity; the flexible GPGPG linker optimizes HTL epitope presentation; the AAY linker facilitates proteasomal cleavage and CTL epitope delivery to MHC-I molecules, ensuring efficient cellular immune responses.

### 2.13 Structural analysis of MEV

First, a Blastp screening against the bovine proteome was conducted to confirm the absence of homologous sequences for MEV. Subsequently, the antigenicity of MEV was predicted using the VaxiJen server (http://www.ddg-pharmfac.net/vaxijen/VaxiJen/VaxiJen.html) (19). The physicochemical properties of the constructed MEV protein, including *in vitro* and *in vivo* half-life, grand average of hydropathicity (GRAVY), aliphatic index (AI), instability index (II), theoretical isoelectric point (pI), and molecular weight (MW), were evaluated using the ProtParam tool on the Expasy server (https://web.expasy.org/protparam/) (23). Finally, the allergenicity of MEV was assessed via the AllerTOP v2.1 server (https://www.ddg-pharmfac.net/AllerTOP/) to ensure that the candidate vaccine does not provoke allergic reactions (20).

### 2.14 Secondary structure prediction of MEV

PDBsum (http://www.ebi.ac.uk/thornton-srv/databases/cgi-bin/pdbsum/GetPage.pl?pdbcode=index.html) was used to predict the secondary structure of the proposed vaccine (24).

### 2.15 Tertiary structure prediction and refinement of MEV

Using the online server I-TASSER (https://zhanglab.ccmb.med.umich.edu/I-TASSER/) modeling the 3D structure of MEV (25). According to the C-scores of structures, the best structure was chosen for further refinement. The side chains of amino acids were repacked to optimize the quality and stability model structures by using the online tool GalaxyRefine (https://galaxy.seoklab.org/) (26). After structure optimization, the optimal tertiary model structure of the vaccine was verified using the PROCHECK module in SAVES v6.0 (https://saves.mbi.ucla.edu/), and the result was shown in the Ramachandran diagram. ProSA-web (https://prosa.services.came.sbg.ac.at/prosa.php) was also employed to obtain the Z score—a parameter representing the rationality of the tertiary model structure (27).

### 2.16 B-cell epitope prediction

For the identification of conformational and linear B-cell epitopes of the designed vaccine, the Ellipro server of the IEDB (https://tools.iedb.org/ellipro/) (28, 29) and ABCPred (https://webs.iiitd.edu.in/raghava/abcpred/ABC_submission.html) (21). The tool was utilized, respectively. The amino-acid sequence of the vaccine was subjected to the ABCPred tool as input; an amino-acid length of 16 was selected, and the selected threshold was 0.5, while the tertiary structure of the vaccine was submitted as input to the Ellipro server with the default parameters selected.

### 2.17 Disulfide engineering

The constructed vaccine model stability needed to be enhanced before further analysis. Disulfide engineering was performed to retain the stability of the designed vaccine construct using Designed 2.0 (http://cptweb.cpt.wayne.edu/DbD2/) (30). The disulfide engineering was performed to prevent the degradation of the vaccine’s weak regions. The detailed structure of the vaccine was submitted and possible residue pairing for disulfide bond engineering based on Chi3 and energy was searched.

### 2.18 Molecular docking

TLR2 (PDB ID: 3A7C) and TLR4 (PDB ID: 4G8A) were used as receptors for molecular docking. Due to the absence of a resolved yak TLR2/TLR4 crystal structure in the PDB database, this selection is justified by: high sequence homology between yak TLRs and the selected receptors (96.2% identity for TLR2, 94.7% identity for TLR4, as confirmed by NCBI BLAST); these structures are the most widely used templates for veterinary TLR docking studies, as documented in multiple published multi-epitope vaccine design papers targeting ruminant species.The molecular docking between the optimized tertiary model structure of MEV and different immune receptors was performed by Cluspro2.0 server (http://cluspro.bu.edu/login.php). Execute: all parameters were maintained at default settings. The receptor structures were prepared by removing water molecules and non-specific ligands. The vaccine construct was treated as a ligand. The lowest energy cluster from 30 generated poses was selected as the optimal binding complex for further analysis. A stable docking structure was selected for each receptor and visualized using PyMol.

### 2.19 Molecular dynamics (MD) simulation

Molecular dynamics (MD) simulations were performed using GROMACS 2024.1 (31) with the Amber14SB (32) force field. The system was solvated in a dodecahedral box with the TIP3P water model (33), and Na^+^/Cl^-^ counterions were added for charge neutralization. Energy minimization was performed with 50,000 steps. A 200-ps NPT equilibration was carried out at 300 K and 1 atm (34).The production simulation ran for 100 ns with a 2 fs time step. RMSD, RMSF, and radius of gyration were calculated to evaluate structural stability.

### 2.20 Immune simulation

*In silico* immune simulations were carried out using the C-ImmSim online server (https://kraken.iac.rm.cnr.it/C-IMMSIM/index.php) (35) with the following parameters: immunization at days 1, 28, and 180; antigen concentration 1000; adjuvant concentration 100; simulation volume 10; random seed 12345; total simulation steps 1080. Note that C-ImmSim is primarily trained on human and murine data, which represents a limitation for yak-specific prediction.

### 2.21 Codon optimization and *In Silico* cloning

Back translation and optimization of the codon usage of the vaccine sequence in E. coli (strain K12) were conducted by JCat (http://www.jcat.de/). Finally, the DNA sequence of the vaccine was inserted between the two sites of the XhoI and EcoRI restriction enzymes in pET28a (+) by the SnapGene tool.

## 3 Results

### 3.1 Protein selection

**Figure 2:**
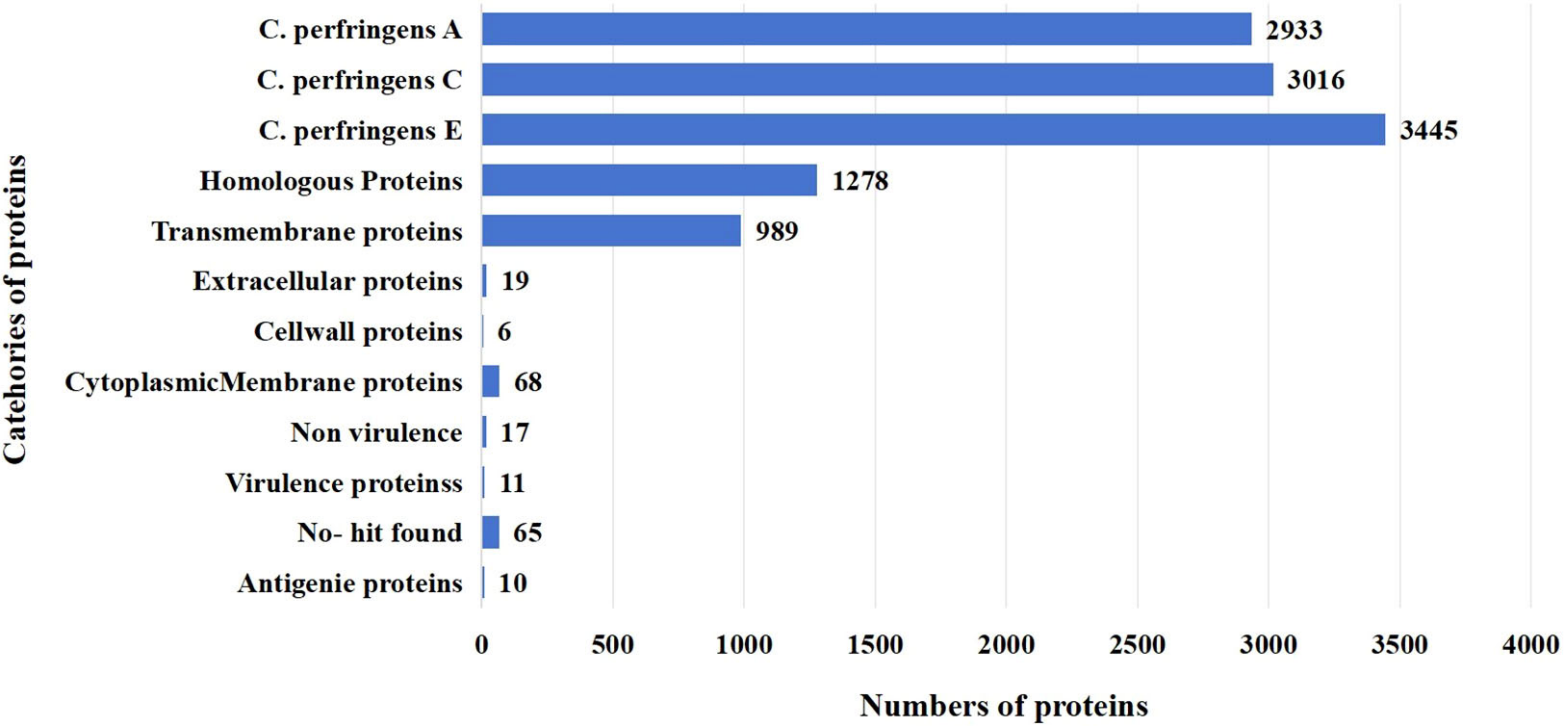
Number of proteins determined in each step of proteome subtraction.

Through whole-genome sequencing analysis, it was determined that type A, C, and E strains contain 2,933 genes (**Supplementary Table 1**), 3,016 genes (**Supplementary Table 2**), and 3,445 genes (**Supplementary Table 3**), respectively. In the retrieval phase, several filters were applied: bovines were considered as hosts, and incomplete proteomes were discarded, resulting in a total of 1,278 homologous proteins of *C. perfringens* derived from yaks (**Supplementary Table 4**). Transmembrane helix analysis was performed using TMHMM-2.0, and proteins with multiple transmembrane helices were discarded, leaving 989 proteins with zero or one transmembrane helix. Subsequently, the online tool PSORTb 3.0.3 was used for subcellular localization, identifying 19 extracellular proteins, 6 cell wall proteins, and 68 cytoplasmic-membrane proteins, resulting in a total of 93 proteins for further analysis. VFDB analysis identified 17 as non-virulent, while 11 were predicted as virulent and considered for subsequent evaluation. Virulent proteins activate strong infection and immunological pathways and are therefore attractive vaccine targets. Finally, the online tool VaxiJen 2.0 was used to evaluate the antigenicity of the screened proteins. Of these, 10 proteins were identified as potential subunit vaccine targets. Finally, the top five highest-ranking virulence proteins—*Iap* (extracellular), *CpsE* (cytoplasmic membrane), *NanH* (extracellular), *Plc* (extracellular), and *Pfo* (extracellular)—were selected as the final candidate vaccine targets, as confirmed in **Table 1**. The number of proteins determined in each step of proteome subtraction is shown in **Figure 2**.

**Table 1:** Candidate proteins and their information.

Gene name	Protein name	UniProtKB Number	Length	Antigenicity score	Subcellular localization
*iap*	Probable endopeptidase p60	Q01835	432	0.6021	Extracellular
*cpsE*	Galactosyl transferase CpsE	Q04664	222	0.5616	Cytoplasmic-Membrane
*nanH*	Sialidase	P10481	382	0.5272	Extracellular
*plc*	Phospholipase C	P0C216	398	0.5186	Extracellular
*pfo*	Perfringolysin O	P0C2E9	500	0.5074	Extracellular

**Figure 3:**
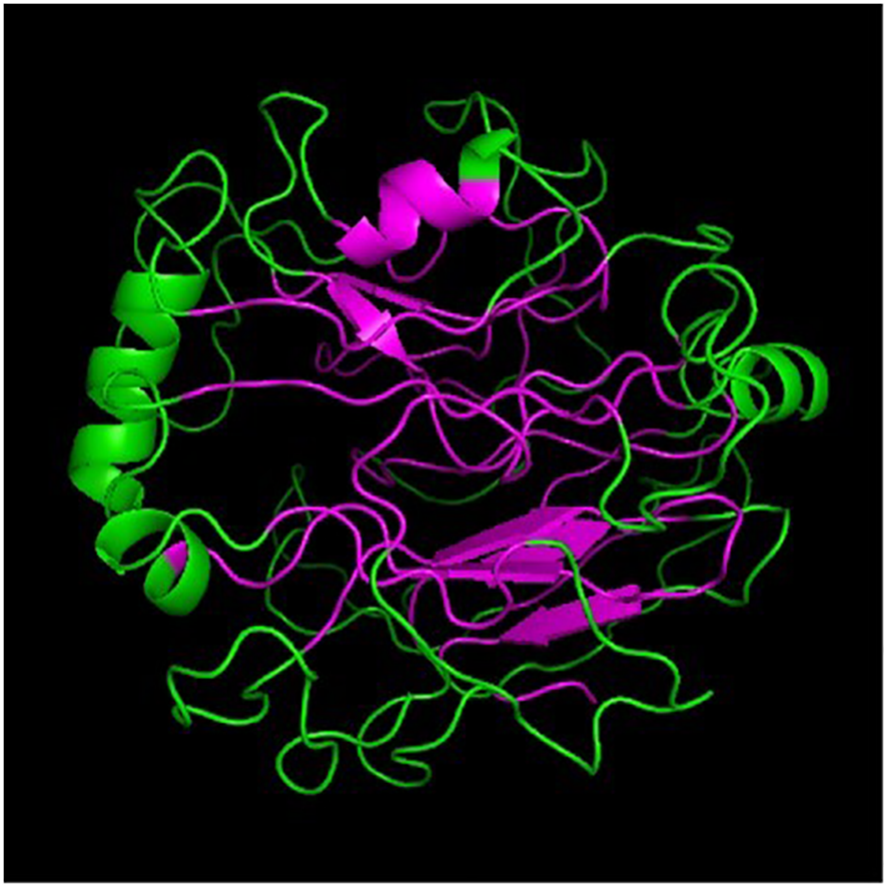
Linear B-cell epitopes (green) are highlighted in the three-dimensional structure of the multi-epitope vaccine (purple).

**Figure 4:**
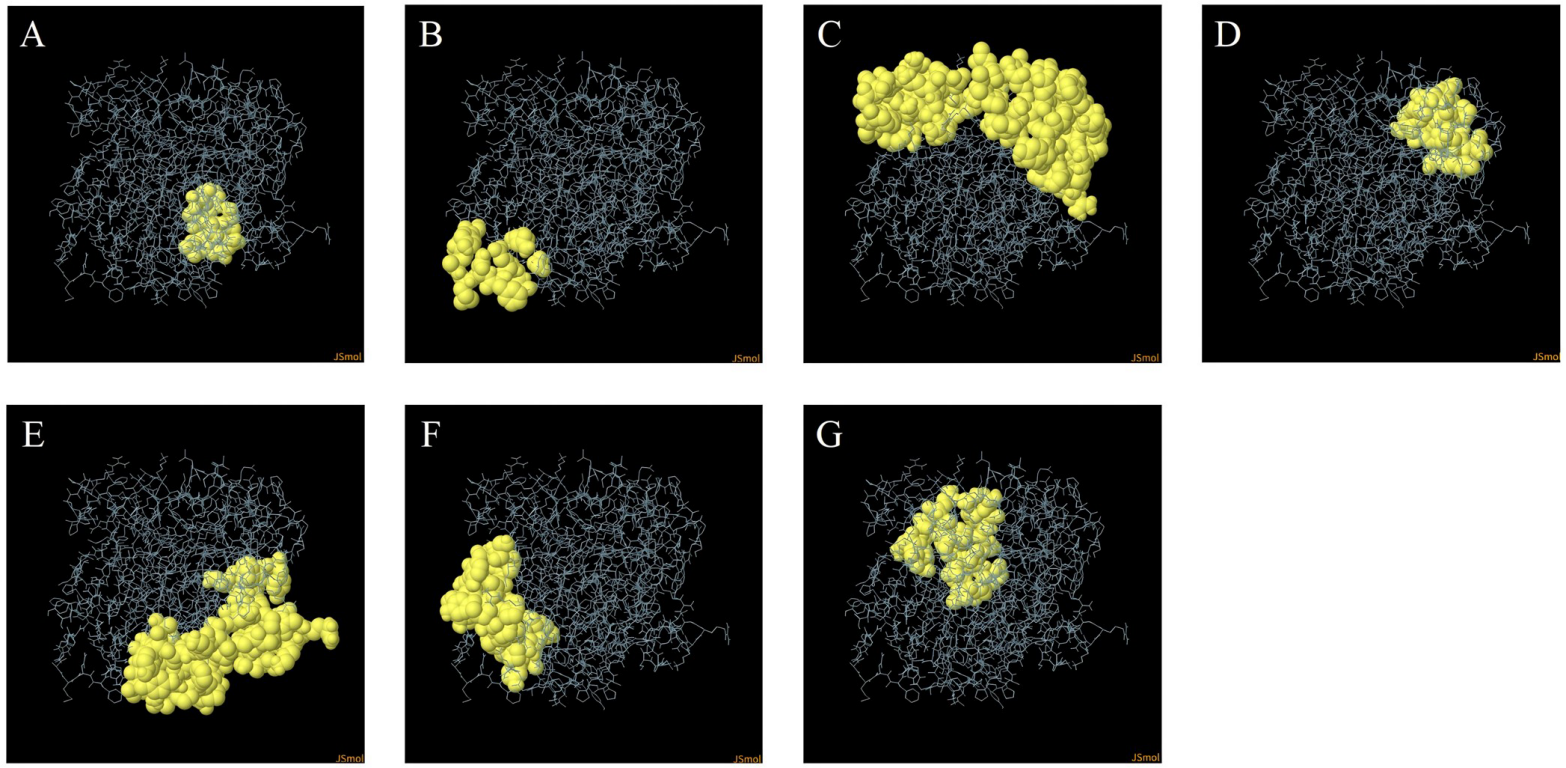
Graphical representation of discontinuous B-cell epitopes on the 3D model of the multi-epitope vaccine **(A–G)**. The vaccine construct and discontinuous B-cell epitopes are depicted in gray sticks and yellow surfaces, respectively.

ABCPred server predicted 32 linear B-cell epitopes with a length of 16 amino acids (**Supplementary Table 7**), which are illustrated in **Figure 3**. The ElliPro server predicted 7 discontinuous B-cell epitopes (**Figure 4**) with the smallest and largest predicted epitopes containing 9 and 65 amino acids, respectively (**Supplementary Table 8**).

### 3.2 Prediction results of T- and B-cell epitopes

Through screening with NetBoLApan-1.0, NetBoLAIIpan-1.0, and ABCpred, 10 CTL epitopes (**Table 2**), 5 HTL epitopes (**Table 3**), and 5 B-cell epitopes (**Table 4**) were predicted and selected from the five candidate proteins. The alleles of CTL epitopes and HTL epitopes are shown in **Supplementary Tables 5** and **6**. These epitopes fulfil all good parameters for epitope-based vaccine design and are non-toxic.

**Table 2:** Screening results of CTL epitopes of candidate proteins.

Proteins	Epitopes	Start position	Length(aa)	Allergenicity	Toxicity	Antigenicity score	No. of Alleles
*Iap*	GQRIRATY	106	8	NP	Non-toxic	1.647	10
	MTFGVGHSV	14	9	NP	Non-toxic	1.0341	15
*cpsE*	SLVGPRPSL	142	9	NP	Non-toxic	1.3423	36
	GQYGKEFNM	71	9	NP	Non-toxic	1.3241	36
*nanH*	RIPNIHLL	37	8	NP	Non-toxic	1.1457	28
	FRIPNIHLL	36	9	NP	Non-toxic	1.4715	23
*Plc*	KENMHELQL	70	9	NP	Non-toxic	0.8647	18
	AKTGKSIYY	225	9	NP	Non-toxic	0.6908	13
*Pfo*	SSKDVQAAF	286	9	NP	Non-toxic	0.9936	37
	YGRTIYVKL	273	9	NP	Non-toxic	1.3793	31

**Table 3:** Screening results of HTL epitopes of candidate proteins.

Proteins	Epitopes	Start position	Length (aa)	Allergenicity	Toxicity	Antigenicity score	No. of Alleles
*Iap*	DINSKIRELEDKIDG	44	15	NP	Non-toxic	1.1401	14
*cpsE*	FLIVAIAIKVEDSDG	47	15	NP	Non-toxic	1.7026	41
*nanH*	WNSKYFRIPNIHLLN	31	15	NP	Non-toxic	0.9189	143
*Plc*	IDTPYHPANVTAVDS	159	15	NP	Non-toxic	0.5221	156
*Pfo*	RKPININIDLPGLKG	126	15	NP	Non-toxic	1.4183	85

**Table 4:** Screening results of B-cell epitopes of candidate proteins.

Proteins	Epitopes	Start position	Length (aa)	Allergenicity	Toxicity	Score	Antigenicity score
*Iap*	DQKQQMEQSQDKYADI	30	16	NP	Non-toxic	0.93	0.9731
*cpsE*	MSGPMFKMKHDPRITK	102	16	NP	Non-toxic	0.89	1.2582
*nanH*	DWSVQMIYSDDNGLTW	134	16	NP	Non-toxic	0.94	0.446
*Plc*	LQLGSTYPDYDKNAYD	76	16	NP	Non-toxic	0.96	0.3035
*Pfo*	VVLGGDAQEHNKVVTK	321	16	NP	Non-toxic	0.96	0.8263

### 3.3 MEV construction

**Figure 5:**
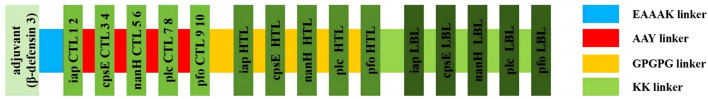
Structure diagram of the MEV.

An MEV was constructed using all selected epitopes. All B cell, HTL, and CTL epitopes were connected through KK, GPGPG, and AAY adapters, respectively. Moreover, human β-defence-3 was selected as an adjuvant. The final structure of the MEV consisted of 352 amino acids, including 1 adjuvant, 10 CTL epitopes, and 5 HTL epitopes. The linkers included one EAAAK linker, nine AAY linkers, five GPGPG linkers, and five KK linkers (**Figure 5**).

### 3.4 Immunogenic and physicochemical analysis

**Figure 6:**
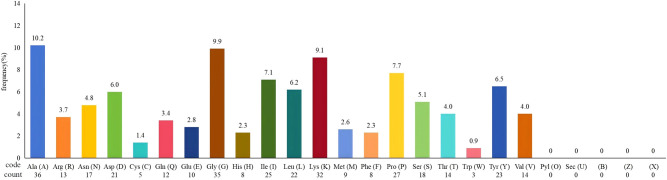
Composition of amino acids in the vaccine construct.

After the vaccine design was completed, a systematic evaluation of its antigenicity and physicochemical properties was conducted (**Table 5**). Antigenicity prediction revealed an antigenicity score of 0.9092 (threshold: 0.5), indicating that the overall structure was determined to be highly antigenic. Assessments for toxicity and allergenicity confirmed that the vaccine is non-toxic and non-allergenic. Analysis of the physicochemical properties of the vaccine using the ProtParam tool showed that the multi-epitope vaccine consists of 352 amino acids, Tryptophan was the least (0.9%), while alanine was the most frequent (10.2%) residue in the vaccine structure (**Figure 6**), with a molecular weight of 38.49 kDa—an ideal size. The theoretical isoelectric point (pI) was calculated to be 9.32. The grand average of hydropathicity (GRAVY) was -0.458, indicating hydrophilic characteristics, which contribute to good solubility in aqueous solutions and facilitate dissolution in physiological buffers for immunization. The instability index was 37.54, classifying the vaccine as a stable protein. The aliphatic index was 73.84, suggesting that the protein maintains good thermal stability even at elevated temperatures. The predicted half-life was within normal ranges for *in vivo* systems in E. coli (>10 h) and *in vitro* systems in mammals (30 h), meeting the requirements for expression and experimental applications.

**Table 5:** Physical and chemical properties of vaccine constructors.

Features	Assessment
Antigenicity score	0.9092
Number of amino acids	352
Theoretical pI	9.32
Molecular weight	38485.06
Total number of negatively charged residues (Asp + Glu)	31
Total number of positively charged residues (Arg + Lys)	45
Formula	C1727H2691N471O499S14
Allergenicity	non-allergen
Instability index	37.54 (stable)
Grand average of hydropathicity (GRAVY)	-0.458
Aliphatic index	73.84
The estimated half-life	30 hours (mammalian reticulocytes, *in vitro*)
	>20 h (yeast, *in vivo*)
	>10 h (*Escherichia coli*, *in vivo*)

### 3.5 Prediction of secondary structure

**Figure 7:**
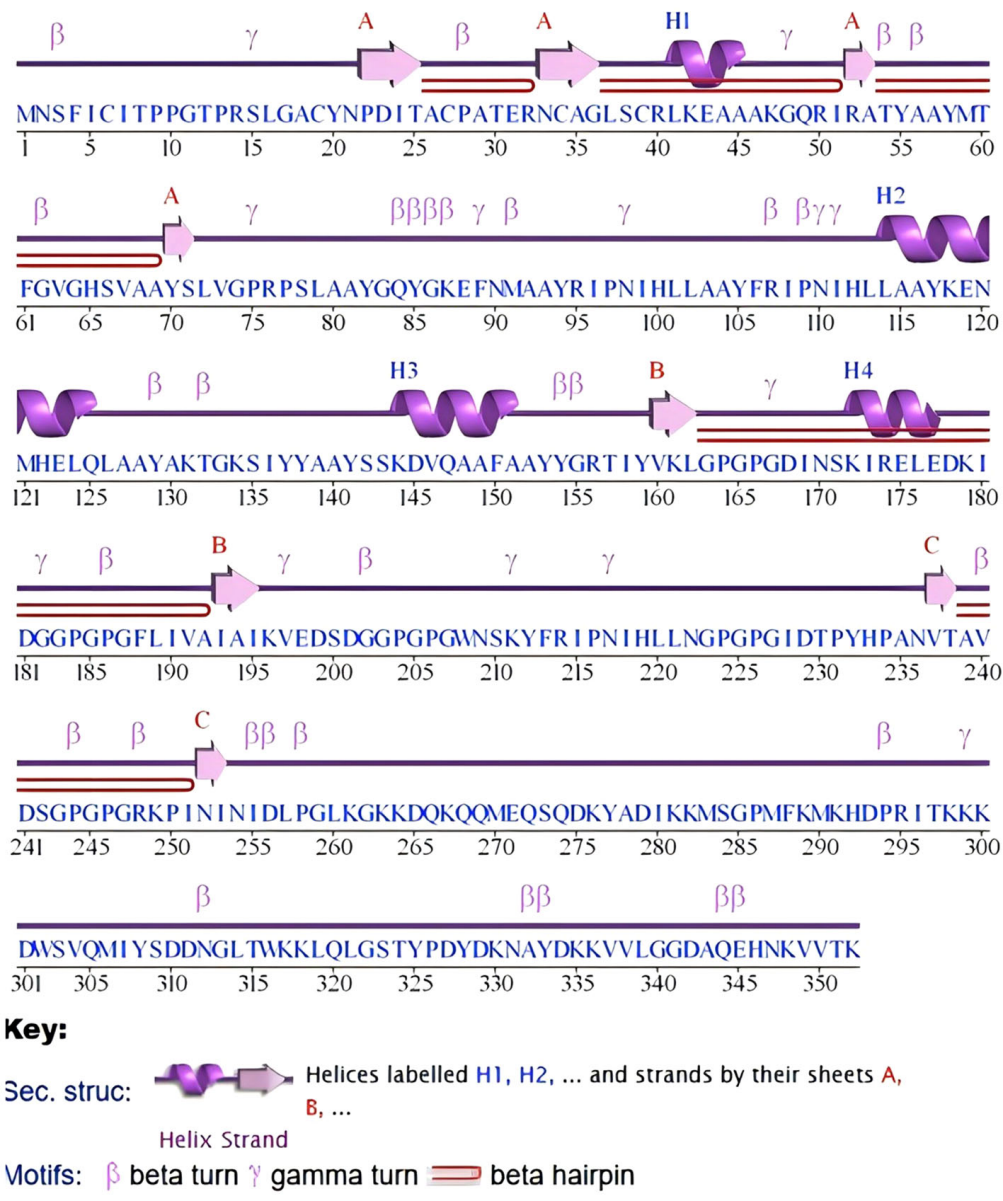
Graphical representation of the secondary structure of the designed vaccine.

The vaccine secondary structure was predicted by the PDBsum. The vaccine construct was composed of 4 helices, 1 helix-helix interaction, 30 beta turns, and 15 gamma turns (**Figure 7**).

### 3.6 Prediction, refinement, and validation of the 3D structure

**Figure 8:**
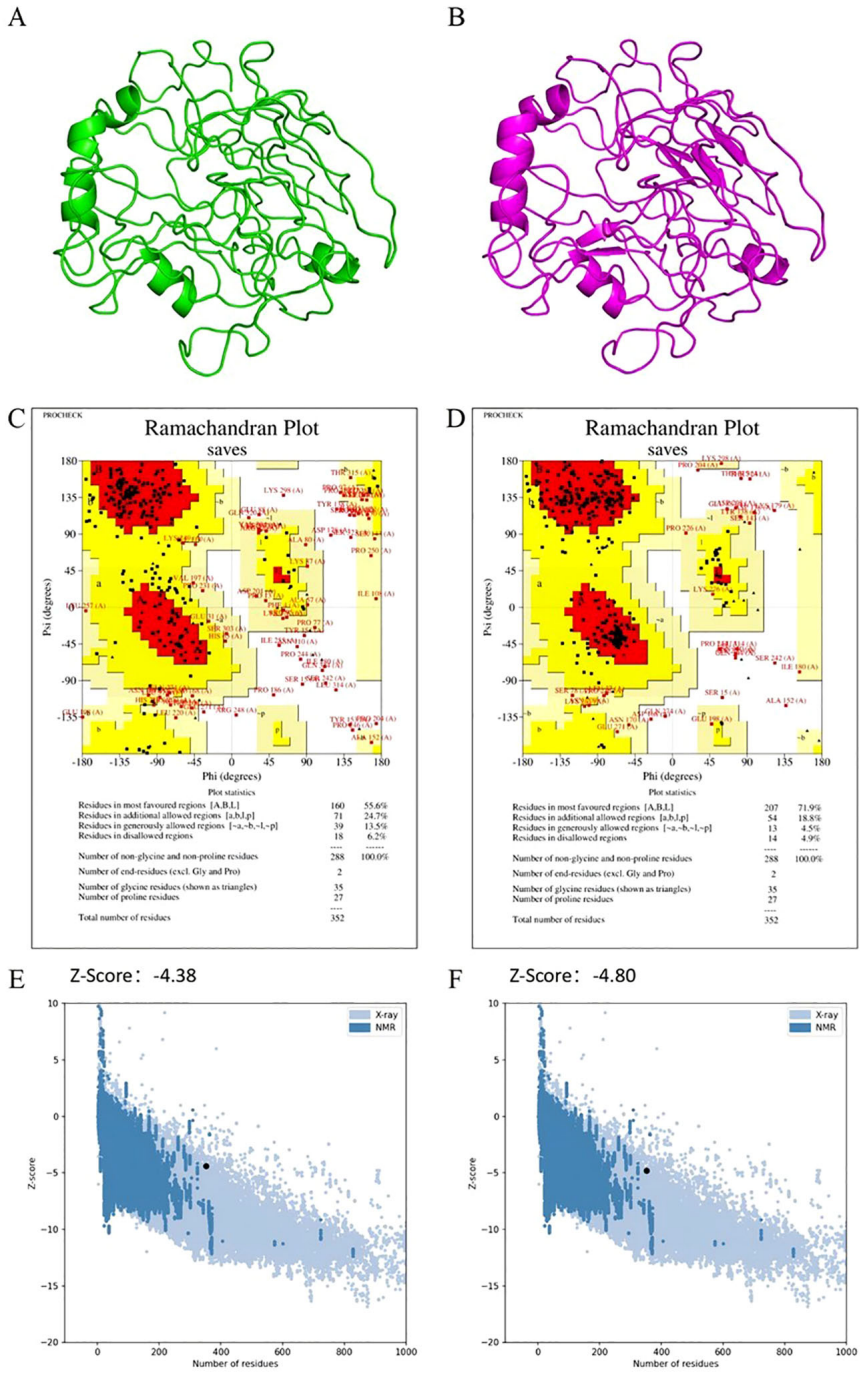
Initial and refined models of the 3D structure of the multi-epitope vaccine. **(A)**, Initial tertiary structure of vaccine Model 1 (C-score = −2.77); **(B)**, Refined tertiary structure of vaccine Model 5 via GalaxyRefine; **(C)**, Ramachandran plot of unrefined model; **(D)**, Ramachandran plot of refined model; **(E)**, ProSA Z-score (−4.38) of unrefined structure; **(F)**, ProSA Z-score (−4.80) of refined structure.

I-TASSER server generated five models for vaccine construct using the threading templates (PDB Hit: 5W71, 2AGS, 7QQKA, 3H71A, and 1DI1). The C-scores of models 1–5 were −2.77, −3.04, −3.30, −3.31, and −3.46, respectively. The C-scores range from −5 to 2 for the predicted 3D models, and a model with the highest C-score is a high-confidence model. Therefore, model 1 (**Figure 8A**) with a C-score of − 2.77 was selected for the refinement. The GalaxyRefine server was used to refine the vaccine structure. The relevant parameters of each model are shown in **Table 6**. After a comprehensive analysis, Model 5 is selected for the next step of analysis (**Figure 8B**). The Ramachandran plot analysis of the unrefined showed that 55.6% of the amino acid residues fell within the most favored regions, 24.7% within the additionally allowed regions, 13.5% within the generously allowed regions, and 6.2% within the disallowed regions (**Figure 8C**). After refinement, 71.9% of the amino acid residues fell within the most favored regions, 18.8% within the additionally allowed regions, 4.5% within the generously allowed regions, and 4.9% within the disallowed regions (**Figure 8D**). The overall quality score for protein structures was obtained from ProSA-web. The Z-score of the unrefined model was calculated to be −4.38 (**Figure 8E**), which reached −4.80 after refinement, suggesting that the optimized tertiary structure model possesses good quality (**Figure 8F**).

**Table 6:** Refined models structure information.

Model	GDT-HA	RMSD	MolProbity	Clash score	Poor	Model
Initial	1.0000	0.000	5.275	379.1	54.1	57.7
MODEL 1	0.9105	0.532	2.978	26.6	2.5	77.7
MODEL 2	0.9020	0.543	2.883	23.9	2.1	77.7
MODEL 3	0.9041	0.541	2.893	23.6	2.1	76.3
MODEL 4	0.9055	0.535	2.762	24.5	1.4	77.4
MODEL 5	0.9162	0.520	2.826	24.1	1.8	77.7

### 3.7 B-cell epitope prediction

ABCPred server predicted 32 linear B-cell epitopes with a length of 16 amino acids (**Supplementary Table 7**), which are illustrated in **Figure 3**. The ElliPro server predicted 7 discontinuous B-cell epitopes (**Figure 4**) with the smallest and largest predicted epitopes containing 9 and 65 amino acids, respectively (**Supplementary Table 8**).

### 3.8 Disulfide engineering

**Figure 9:**
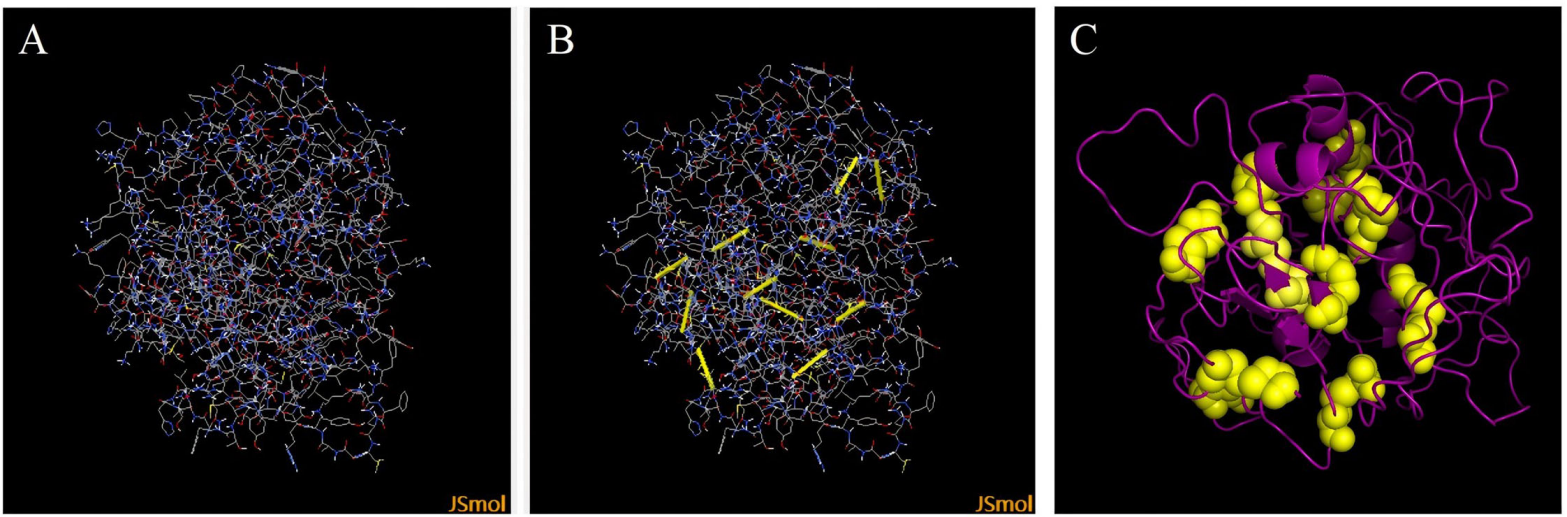
Wild structure of the vaccine **(A)** and mutated structure of the vaccine **(B, C)**.

The protein was subjected to disulfide engineering, which involved the replacement of 11 amino acid residues prone to enzymatic degradation. The targeted residues were replaced by cysteine amino acid as represented by yellow sticks in the mutated structure (**Figures 9A, B**). Additionally, the replaced amino acids are also represented by spheres in the 3D structure (**Figure 9C**). The pairs of amino acid residues and their chi3 energy value obtained during disulfide engineering are tabulated in **Supplementary Table 9**.

### 3.9 Molecular docking analysis

**Figure 10:**
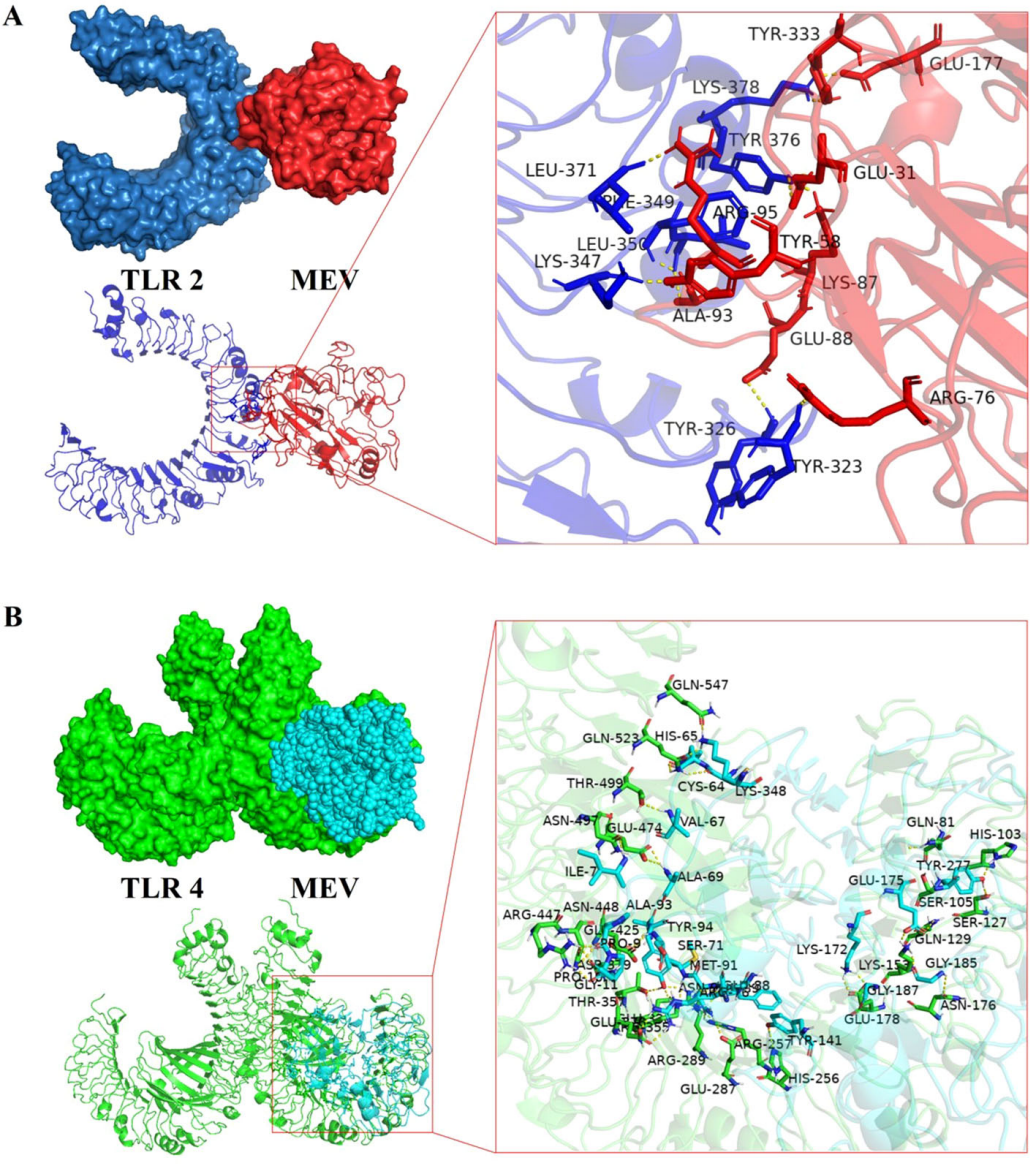
Molecular docking of multi-epitope vaccines with TLR2 **(A)** and TLR4 **(B)**.

Molecular docking analysis was performed to check the binding affinity of the vaccine construct to immune cell receptors TLR2 and TLR4. For docking purposes, Cluspro2.0 was utilized; in each case, the 30 docking solutions were generated. The results were interpreted through binding energy; the solution that has the lowest binding energy value demonstrates stronger intermolecular binding affinity. The docked complexes, which have the lowest binding energy, were selected for interaction visualization analysis. All 30 generated docked solutions for each receptor and their binding energy are tabulated in **Supplementary Tables 10** and **11**. The best docked solution in each case was considered for molecular dynamics analysis. The intermolecularly docked complexes are presented in a 3D structure in **Figure 10**. The binding energy value of the vaccine with TLR2 and TLR4 is −1024.6 kcal/mol and −1104.4 kcal/mol, respectively. Molecular docking results showed that the multi-epitope vaccine bound to the biologically active pockets of TLR2 and the TLR4-MD2 interface, which are critical for receptor dimerization and downstream signaling. The vaccine interacted with key functional residues within these binding regions: for TLR2, the interacting residues (Y326, Y376, L350, F349, as listed in **Supplementary Table 12**) are all located in the extracellular LRR domain, the core active pocket for ligand recognition; for TLR4, the interacting residues (H256, R447, R335, N176, E474, Q523, T499, E425, Q129, N497, D379, R289, E287, R355, Q81, as listed in **Supplementary Table 13**) are all located in the TLR4-MD2 functional interface, confirming the biological validity of the docking poses. Hydrogen bond analysis was re-evaluated after energy minimization and structural optimization using the AMBER ff14sb forcefield with TIP3P water model and 0.15 M NaCl. All stable hydrogen bonds in the **Supplementary Table 12** fall within the standard range of 2.5–2.6 Å for protein structures. The hydrogen bonds in the **Supplementary Table 13** were all within 2.0–3.0 Å, and were further optimized to the **Supplementary Tables 2.5**-**3.0** Å range, verifying the structural stability and rationality of the vaccine-receptor complexes.

### 3.10 MD simulation

To evaluate the dynamic stability of the vaccine–TLR complexes, we performed 100 ns molecular dynamics simulations combined with multiple analytical methods. The TLR4–vaccine complex exhibited overall superior structural stability compared with the TLR2–vaccine complex.

**Figure 11:**
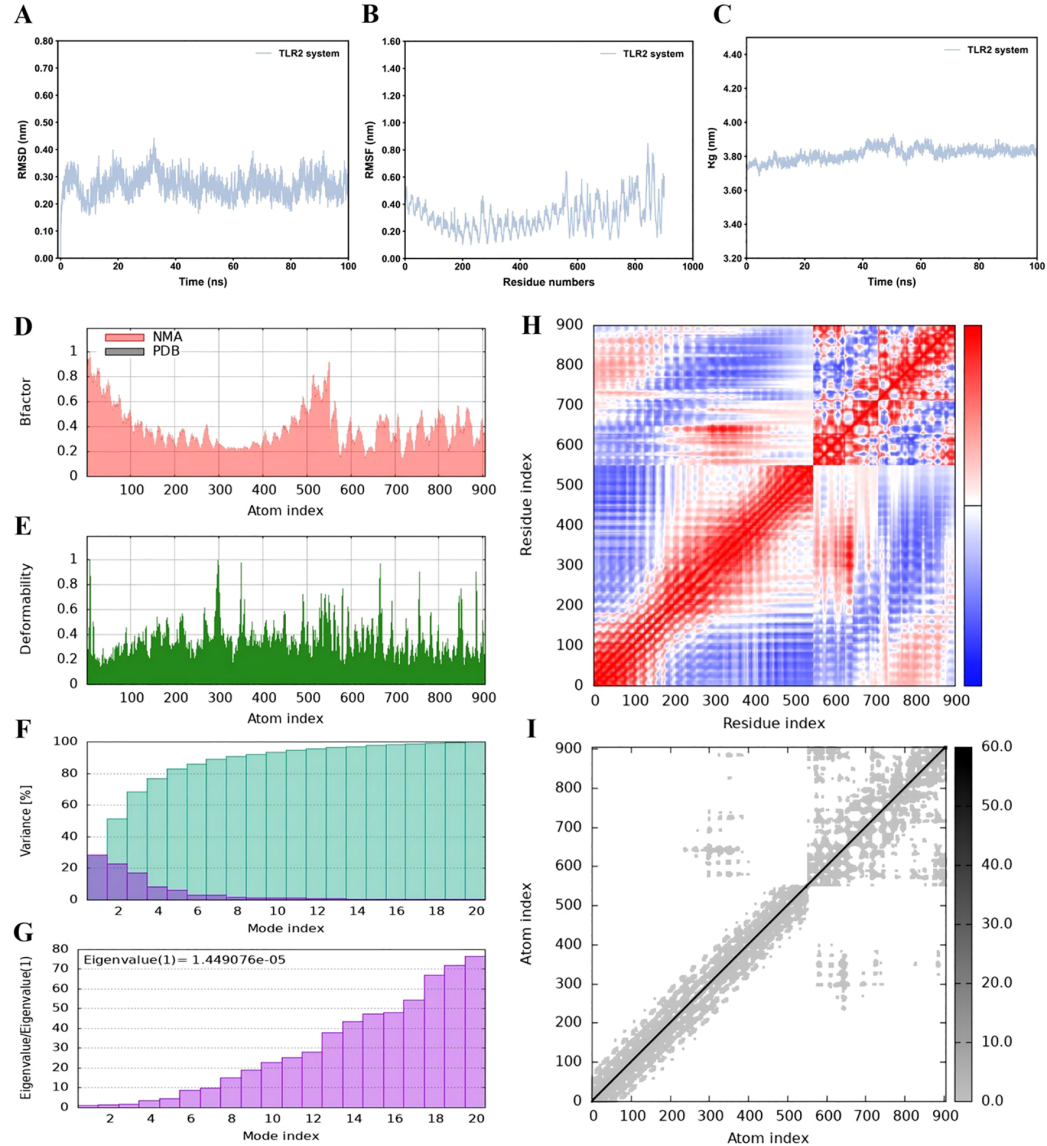
Molecular dynamic (MD) simulation results of the MEV-TLR2 complex. **(A–C)** MD simulation of the vaccine-TLR2 complex, including the RMSD value of the complex backbone, the RMSF value of side-chain residues, and the radius of gyration during the molecular dynamic simulation. **(D–I)** Results of iMODS of vaccine-TLR4 docking complex; **(D)**, B-factor; **(E)**, Deformability plot; **(F)**, Variance; **(G)**, Eigenvalue; **(H)**, Covariance matrix analysis; **(I)**, Elastic network model.

**Figure 12:**
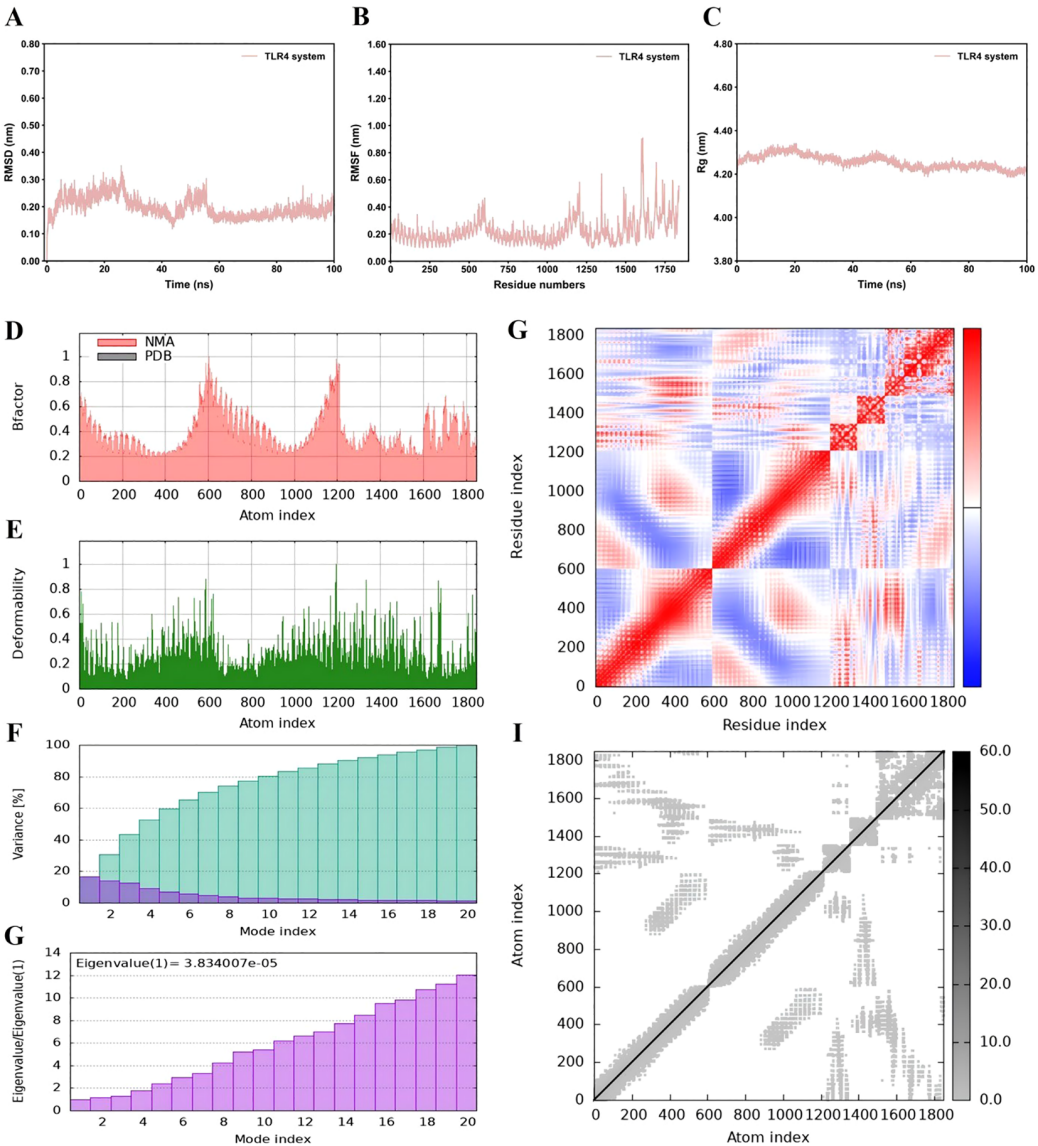
Molecular dynamic (MD) Simulation Results of the MEV-TLR4 Complex. **(A–C)** MD simulation of the vaccine-TLR4 complex, including the RMSD value of the complex backbone, the RMSF value of side-chain residues, and the radius of gyration during the molecular dynamic simulation. **(D–I)** Results of iMODS of vaccine-TLR4 docking complex; **(D)**, B-factor; **(E)**, Deformability plot; **(F)**, Variance; **(G)**, Eigenvalue; **(H)**, Covariance matrix analysis; **(I)**, Elastic network model.

In terms of global conformational stability, RMSD analysis (**Figures 11A**, **12A**) showed that the TLR4–vaccine complex had an average RMSD of 0.1971 ± 0.0377 nm, which converged rapidly after 60 ns and remained stable with minimal fluctuations. In contrast, the TLR2–vaccine complex displayed a higher average RMSD (0.2692 ± 0.0420 nm) and greater fluctuations, indicating lower conformational stability. RMSF analysis (**Figures 11B**, **12B**) further revealed that the TLR2–vaccine complex exhibited pronounced fluctuation peaks in multiple regions, while the TLR4–vaccine complex showed overall lower and smoother fluctuations, reflecting greater structural rigidity. Regarding structural compactness (**Figures 11C**, **12C**), the radius of gyration (Rg) of the TLR4–vaccine complex was 4.2562 ± 0.0306 nm, with very little variation (SD = 0.0306 nm), suggesting a highly compact and stable conformation. Although the TLR2–vaccine complex had a smaller Rg (3.8152 ± 0.0391 nm), it showed greater variability, indicating weaker overall rigidity.

Further normal mode analysis (**Figures 11D–I**, **12D–I**) revealed that the TLR4–vaccine complex had lower overall B-factor values, lower deformability, and a variance distribution indicative of highly correlated motions. Its eigenvalue (3.834 × 10^-5^) was significantly higher than that of the TLR2 complex (1.042 × 10^-5^), suggesting stronger structural rigidity and more coordinated movements. Covariance matrix and elastic network model analyses also demonstrated more extensive and continuous strong interaction networks between residues in the TLR4 complex, supporting its higher overall stability.

In summary, all analytical metrics consistently indicate that the TLR4–vaccine complex possesses superior structural rigidity, conformational stability, and internal coordination in dynamic simulations, suggesting stronger binding stability.

### 3.11 Immune simulation

**Figure 13:**
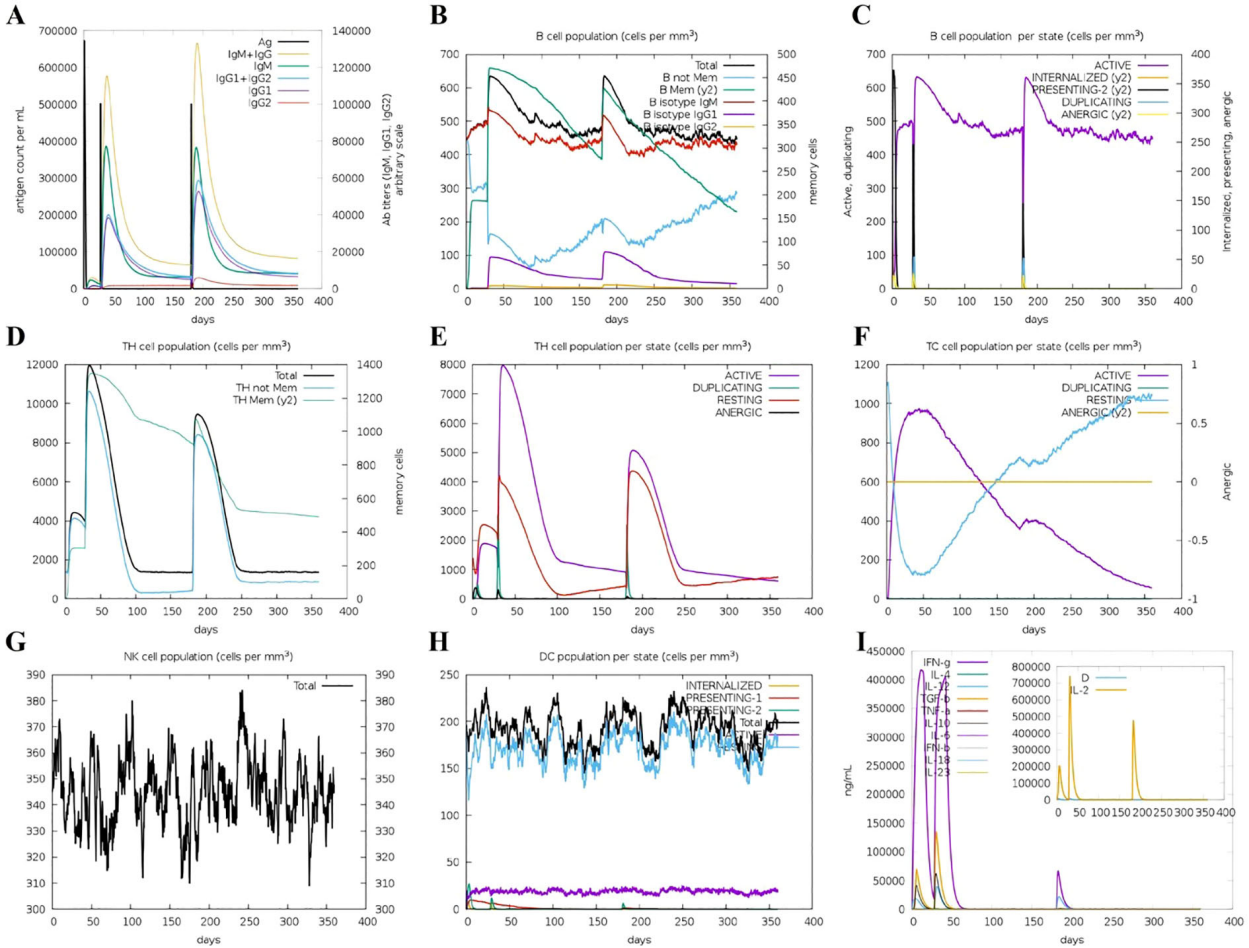
*In silico* immune simulation profiles of the multi-epitope vaccine. Simulations were performed using the C-ImmSim server with a single random seed (seed=12345), which may introduce minor computational artifacts. **(A)**, Antigen (Ag) clearance and antibody production. **(B)**, Total B cell and B memory cell counts. **(C)**, B-cell population states. **(D)**, Total Helper T-cell (TH) and TH memory cell counts. **(E)**, Helper T-cell population states. **(F)**, Cytotoxic T-cell (TC) population states. **(G)**, Natural killer (NK) cell count. **(H)**, Dendritic cell (DC) population states. **(I)**, Cytokine levels.

To evaluate the immune response elicited by the designed multi-epitope vaccine, we performed *in silico* simulation using the C-ImmSim server. The results (**Figure 13**) demonstrated that the vaccine effectively induced a comprehensive and durable adaptive immune response. Following the introduction of the vaccine antigen, its concentration decreased rapidly and was cleared quickly, while the titer of specific antibodies increased significantly, indicating the successful triggering of a potent humoral immune response (**Figure 13A**). Analysis of the cellular immune response revealed that the vaccine stimulated the activation and proliferation of different lymphocyte subsets: the populations of B cells (**Figure 13B**), helper T cells (**Figure 13C**), and cytotoxic T cells (**Figure 13D**) all exhibited prominent peaks after antigen exposure, followed by successful differentiation into memory cells, suggesting the potential for long-term immune memory. Furthermore, innate immune cells such as natural killer cells (**Figures 13E, G**) and dendritic cells (**Figure 13F, H**) were also strongly activated, indicating an effective bridge between innate and adaptive immune responses. Crucially, cytokine profile analysis (**Figure 13I**) showed substantial secretion of Th1-type cytokines, including interleukin-2 (IL-2) and interferon-gamma (IFN-γ), which are indicative of robust cell-mediated immunity, further confirming the vaccine’s propensity to elicit a potent immune response dominated by cellular immunity.

### 3.12 Codon adaptation and *In Silico* cloning

**Figure 14:**
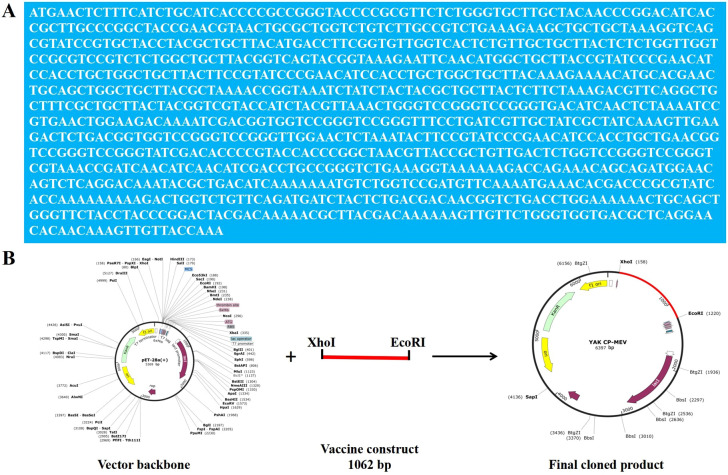
*In silico* cloning of the vaccine construct in the pET28a (+). The vaccine’s gene sequence is shown in red, and the backbone of the pET28a (+) is shown in black. **(A)**, Optimized vaccine codon sequence for E. coli K12; **(B)**, Schematic of inserting vaccine fragment into pET28a (+) between XhoI and EcoRI sites.

Back translation and optimization of the codon usage of the vaccine sequence in *E. coli* (strain K12) were conducted by JCat (**Figure 14A**). The GC content and CAI value of the vaccine construct were calculated as 51.4% and 1, respectively. Finally, the DNA sequence of the vaccine was inserted between the two sites of the XhoI and EcoRI restriction enzymes in pET28a (+) by the SnapGene tool (**Figure 14B**).

## 4 Discussion

*C. perfringens* is a critical zoonotic pathogen and the primary causative agent of intestinal toxemia in yaks. Currently, commercial prophylactic vaccines against *C. perfringens* primarily rely on inactivated formulations. There is an urgent need to develop multi-epitope vaccines to combat *C. perfringens* infections in yaks and curb the progression of related diseases. Reverse vaccinology, a computational approach to vaccine design, has emerged as a powerful tool in recent years (36). This methodology has been successfully applied to design novel vaccines against various pathogens, including Streptococcus (36), Brucella (37), Staphylococcus (38), Rotavirus (38), Monkeypox virus (39), HPV (40), and Toxoplasma gondii (41). The application of MEV design has also driven notable advances in veterinary vaccine development. However, despite these advances, a critical gap remains in the development of vaccines specifically tailored for yaks on the Qinghai-Tibet Plateau.

In the current landscape of *C. perfringens* vaccine research, conventional approaches predominantly rely on single toxoid vaccines (e.g., Type A epsilon toxoid) or polyvalent bacterins. While these have been staples in livestock management, they often exhibit limited cross-protection against the diverse toxinotypes (A, C, and E) prevalent in yaks and suffer from variable efficacy due to species-specific immune responses. Recent recombinant subunit vaccines have attempted to address these issues, yet few integrate a multi-epitope design capable of simultaneously targeting conserved virulence factors across multiple strains. In this study, we employed an integrated immunoinformatics pipeline to construct a novel vaccine candidate incorporating epitopes from multiple prevalent toxinotypes (A, C, E) of *C. perfringens* affecting yaks, thereby overcoming the limitations of conventional vaccines that typically target a single strain or toxin type. Rigorous computational validation, including structural analysis, molecular docking, MD simulations, and immune profiling, provides compelling evidence for the vaccine’s potential efficacy and stability, offering new strategies for preventing *C. perfringens* disease in yaks.

The vaccine was designed by selecting conserved and immunogenic epitopes from five core virulence factors: *Iap*, *CpsE*, *NanH*, *Plc*, and *Pfo*. This multi-toxinotype approach enables broad-spectrum coverage against the major toxinotypes impacting yaks, which is particularly vital in regions such as the Qinghai-Tibet Plateau, where co-infections or circulating diverse strains are common. The inclusion of both T-cell and B-cell epitopes ensures comprehensive activation of complementary adaptive immune pathways. For instance, T-cell epitopes are anticipated to induce robust cellular immunity, while linear B-cell epitopes are likely to drive the production of neutralizing antibodies (42). Furthermore, the strategic use of appropriate linkers (AAY, GPGPG, KK) enhances the stability of the vaccine construct and promotes optimal epitope processing and presentation. A key innovation in this design is the incorporation of human β-defensin-3 as an adjuvant. This molecule functions as a TLR4 agonist, potentiating innate immune activation and facilitating dendritic cell maturation (43). Immune simulation results (**Figure 13**) support this mechanism, showing elevated levels of IL-2 and IFN-γ, indicative of a Th1-skewed response, which is essential for combating intracellular pathogens (44). The balanced Th1/Th2 profile observed in simulations further suggests a reduced risk of vaccine-associated immunopathology.

Physicochemical analyses confirmed optimal characteristics: a molecular weight of approximately 38.5 kDa, an instability index of 37.54 (indicating stability), and a hydrophilic nature (GRAVY: -0.458). These properties support high solubility and facilitate efficient production in prokaryotic expression systems, as demonstrated by successful *in silico* cloning into the pET-28a (+) vector. Secondary structure predictions revealed a well-balanced composition of α-helices, β-turns, and strands, which was further refined to improve the tertiary structure. The refined model exhibited significant enhancement in Ramachandran plot statistics, with 71.9% of residues residing in favored regions, and a ProSA Z-score of -4.80, confirming the model’s high reliability. This study systematically evaluated the interaction characteristics between the vaccine protein and Toll-like receptors TLR2 and TLR4 using molecular docking, MD simulations, and normal mode analysis (NMA). Molecular docking results indicated that the vaccine can form stable complexes with both receptors. The interaction with TLR2 is stabilized by a hydrogen bond network and hydrophobic interactions involving residues such as GLU-177 and TYR-333 from the vaccine and LYS-378 and TYR-376 from TLR2. In contrast, the vaccine–TLR4 interaction is more extensive, involving residues such as LYS-348 and GLU-175 from the vaccine and GLN-81 and HIS-179 from TLR4, suggesting superior interface complementarity. MD simulations further revealed that the TLR4–vaccine complex exhibits higher dynamic stability over 100 ns. Its RMSD value (0.1971 ± 0.0377 nm) was notably lower than that of the TLR2 complex (0.2692 ± 0.0420 nm), with faster convergence and smaller fluctuations. RMSF analysis indicated higher overall flexibility in the TLR2 complex, while the TLR4 complex displayed greater conformational rigidity. Rg analysis showed that although the TLR4 complex displayed a larger Rg value due to its bigger size, its fluctuation (SD = 0.0306 nm) was lower than that of the TLR2 complex (SD = 0.0391 nm), reflecting better conformational compactness. NMA results were consistent with the MD findings. The TLR4–vaccine complex exhibited a smoother B-factor distribution, lower deformability peaks, and a significantly higher eigenvalue (3.834 × 10^-5^), compared with the TLR2 complex (1.042 × 10^-5^)—indicating stronger overall rigidity and more coordinated motion. The covariance matrix analysis showed broader correlated motions between residues in the TLR4 complex, and the elastic network model confirmed more intensive internal interaction networks. Multi-dimensional computational analyses demonstrate that the vaccine–TLR4 complex possesses superior structural stability, rigidity, and dynamic coordination compared to the vaccine–TLR2 complex.

C-ImmSim simulations projected a comprehensive immune response characterized by rapid antigen clearance, potent antibody production (IgM and IgG), and memory cell formation (**Figures 13A–D**). Robust clonal expansion of B and T cells was observed following each immunization, accompanied by sustained memory cell populations. Activation of innate immune components (e.g., NK cells and dendritic cells) effectively bridged the adaptive immune response (45). The cytokine profile was dominated by Th1-type cytokines (IFN-γ and IL-2), supporting the induction of cell-mediated immunity. These results suggest that the vaccine could confer long-term protection, which is crucial in endemic areas with frequent pathogen exposure. Although the calculation results are promising, experimental validation is crucial. In future work, it is necessary to confirm TLR4 binding and cytokine induction through *in vitro* testing. Conduct animal challenge studies in yak or mouse models to evaluate the protective effect on heterologous *C. perfringens* strains. This study underscores the power of immunoinformatics in addressing veterinary health challenges. The pipeline established herein, from genomic mining to immune simulation, provides a reproducible framework for developing vaccines against other pathogens. The vaccine design also incorporates practical considerations, such as codon optimization for high-yield expression in *E. coli*, facilitating future large-scale production.

Collectively, the *in silico* results suggest that the multi-epitope vaccine may serve as a promising candidate against *C. perfringens* in yaks. The primary breakthrough of this design lies in its transition from conventional single-target approaches to a broad-spectrum strategy, specifically addressing the critical gap in cross-protection for co-infections prevalent on the Qinghai-Tibet Plateau. While these predictions require further experimental validation, this work establishes a foundational blueprint for a next-generation vaccine that moves beyond the limitations of current inactivated vaccines, providing a targeted, stable, and broadly protective solution for yak health management.

## 5 Limitations and future perspectives

This study was conducted using an *in silico* immunoinformatics approach, which provides a rapid and rational framework for multi-epitope vaccine design. As with most computational vaccine studies, the present work has a few limitations. First, all predictions are theoretical and cannot fully account for *in vivo* factors such as post-translational modifications that may mask epitopes, so further *in vitro* and *in vivo* validation is essential. Second, epitope prediction and molecular docking relied on well-conserved bovine and human templates due to the current lack of yak-specific MHC and TLR structures, which are standard practices in veterinary immunoinformatics research. Despite these limitations, our results support the potential of this multi-epitope vaccine as a promising candidate against yak *C. perfringens*. Future work will focus on experimental verification of its immunogenicity and protective efficacy, including *in vitro* yak peripheral blood mononuclear cell (PBMC) stimulation assays to evaluate antigen-specific immune responses, and *in vivo* immunization and challenge trials to confirm its actual protective potential against *C. perfringens* infection in yaks.

## 6 Conclusion

The designed multi-epitope vaccine represents a computationally validated strategy for combating *C. perfringens* infections in yaks. By integrating epitopes from multiple toxinotypes and incorporating immune-enhancing adjuvants, the vaccine achieves broad-spectrum potential while ensuring safety and stability. Strong receptor binding, favorable dynamics, and robust immune simulations support its prospects as a viable vaccine candidate. This work provides a foundation for experimental development and highlights the power of immunoinformatics in addressing veterinary health challenges.

## Data Availability

The datasets presented in this study can be found in online repositories. The names of the repository/repositories and accession number(s) can be found in the article/**Supplementary Material**.

## Supplementary material

Supplementary Table 1 Genome of type A *Clostridium perfringens*.

Supplementary Table 2 Genome of type C *Clostridium perfringens*.

Supplementary Table 3 Genome of type E *Clostridium perfringens*.

Supplementary Table 4 Homologous proteins of *Clostridium perfringens* derived from yaks.

Supplementary Table 5 Alleles of CTL epitopes.

Supplementary Table 6 Alleles of HTL epitopes.

Supplementary Table 7 Linear B-cell epitopes of the multi-epitope vaccine.

Supplementary Table 8 Discontinuous B-cell epitopes of the multi-epitope vaccine.

Supplementary Table 9 Pairs of amino acid residues and their chi3 energy value obtained during disulfide engineering.

Supplementary Table 10 Docking score of vaccine with TLR2.

Supplementary Table 11 Docking score of vaccine with TLR4.

Supplementary Table 12 List of amino acids involved in the formation of hydrogen bonds between TLR2 and the vaccine construct.

Supplementary Table 13 List of amino acids involved in the formation of hydrogen bonds between TLR4 (chain A) and the vaccine construct.

## References

[1] Li A Liu C Han X Zheng J Zhang G Qi X . Tibetan Plateau yak milk: A comprehensive review of nutritional values, health benefits, and processing technology. Food Chem X. (2023) 20:100919. doi: 10.1016/j.fochx.2023.100919 38144800 PMC10739763

[2] Wu D Luo R Gong G Zhang L Huang J Cai C . Antimicrobial susceptibility and multilocus sequence typing of Clostridium perfringens isolated from yaks in Qinghai-Tibet plateau, China. Front Vet Sci. (2022) 9:1022215. doi: 10.3389/fvets.2022.1022215 36325097 PMC9619089

[3] Hassan KA Elbourne LDH Tetu SG Melville SB Rood JI Paulsen IT . Genomic analyses of Clostridium perfringens isolates from five toxinotypes. Res Microbiol. (2015) 166:255–63. doi: 10.1016/j.resmic.2014.10.003 25445567

[4] Rood JI Adams V Lacey J Lyras D McClane BA Melville SB . Expansion of the Clostridium perfringens toxin-based typing scheme. Anaerobe. (2018) 53:5–10. doi: 10.1016/j.anaerobe.2018.04.011 29866424 PMC6195859

[5] Camargo A Ramírez JD Kiu R Hall LJ Muñoz M . Unveiling the pathogenic mechanisms of Clostridium perfringens toxins and virulence factors. Emerg Microbes Infect. (2024) 13:2341968. doi: 10.1080/22221751.2024.2341968 38590276 PMC11057404

[6] Navarro MA McClane BA Uzal FA . Mechanisms of action and cell death associated with Clostridium perfringens toxins. Toxins (Basel). (2018) 10:212. doi: 10.3390/toxins10050212 29786671 PMC5983268

[7] Li C Zhu Y Qi X Chai Z Luo J Shang K . Design of a multi-epitope recombinant BCG vaccine targeting Brucella OMP31, LptE and VirB2 in immunoinformatics approaches. PloS One. (2025) 20:e0334843. doi: 10.1371/journal.pone.0334843 41196893 PMC12591482

[8] Momajadi L Khanahmad H Mahnam K . Designing a multi-epitope influenza vaccine: an immunoinformatics approach. Sci Rep. (2024) 14:25382. doi: 10.1038/s41598-024-74438-w 39455641 PMC11512060

[9] Soto LF Romaní AC Jiménez-Avalos G Silva Y Ordinola-Ramirez CM Lopez Lapa RM . Immunoinformatic analysis of the whole proteome for vaccine design: an application to Clostridium perfringens. Front Immunol. (2022) 13:942907. doi: 10.3389/fimmu.2022.942907 36110855 PMC9469472

[10] Oli AN Obialor WO Ifeanyichukwu MO Odimegwu DC Okoyeh JN Emechebe GO . Immunoinformatics and vaccine development: an overview. ImmunoTargets Ther. (2020) 9:13–30. doi: 10.2147/ITT.S241064 32161726 PMC7049754

[11] Huang Y Niu B Gao Y Fu L Li W . CD-HIT Suite: a web server for clustering and comparing biological sequences. Bioinformatics. (2010) 26:680–2. doi: 10.1093/bioinformatics/btq003 20053844 PMC2828112

[12] Lavigne R Seto D Mahadevan P Ackermann HW Kropinski AM . Unifying classical and molecular taxonomic classification: analysis of the Podoviridae using BLASTp-based tools. Res Microbiol. (2008) 159:406–14. doi: 10.1016/j.resmic.2008.03.005 18555669

[13] Wen QF Liu S Dong C Guo HX Gao YZ Guo FB . Geptop 2.0: an updated, more precise, and faster Geptop server for identification of prokaryotic essential genes. Front Microbiol. (2019) 10:1236. doi: 10.3389/fmicb.2019.01236 31214154 PMC6558110

[14] Ahmad S Azam SS . A novel approach of virulome based reverse vaccinology for exploring and validating peptide-based vaccine candidates against the most troublesome nosocomial pathogen: Acinetobacter baumannii. J Mol Graph Model. (2018) 83:1–11. doi: 10.1016/j.jmgm.2018.04.020 29753164

[15] Ismail S Shahid F Khan A Bhatti S Ahmad S Naz A . Pan-vaccinomics approach towards a universal vaccine candidate against WHO priority pathogens to address growing global antibiotic resistance. Comput Biol Med. (2021) 136:104705. doi: 10.1016/j.compbiomed.2021.104705 34340127

[16] Yu NY Wagner JR Laird MR Melli G Rey S Lo R . PSORTb 3.0: improved protein subcellular localization prediction with refined localization subcategories and predictive capabilities for all prokaryotes. Bioinformatics. (2010) 26:1608–15. doi: 10.1093/bioinformatics/btq249 20472543 PMC2887053

[17] Chen L Yang J Yu J Yao Z Sun L Shen Y . VFDB: a reference database for bacterial virulence factors. Nucleic Acids Res. (2005) 33:D325–8. doi: 10.1093/nar/gki008 15608208 PMC539962

[18] Larsen MV Lundegaard C Lamberth K Buus S Lund O Nielsen M . Large-scale validation of methods for cytotoxic T-lymphocyte epitope prediction. BMC Bioinform. (2007) 8:424. doi: 10.1186/1471-2105-8-424 17973982 PMC2194739

[19] Doytchinova IA Flower DR . VaxiJen: a server for prediction of protective antigens, tumour antigens and subunit vaccines. BMC Bioinform. (2007) 8:4. doi: 10.1186/1471-2105-8-4 17207271 PMC1780059

[20] Dimitrov I Bangov I Flower DR Doytchinova I . AllerTOP. J Mol Model. (2014) 20:2278. doi: 10.1186/1471-2105-14-s6-s4 24878803

[21] Zheng D Liang S Zhang C . B-cell epitope predictions using computational methods. Methods Mol Biol. (2023) 2552:239–54. doi: 10.1007/978-1-0716-2609-2_12 36346595

[22] Chen X Zaro JL Shen WC . Fusion protein linkers: property, design and functionality. Adv Drug Delivery Rev. (2013) 65:1357–69. doi: 10.1016/j.addr.2012.09.039 23026637 PMC3726540

[23] Gasteiger E Gattiker A Hoogland C Ivanyi I Appel RD Bairoch A . Expasy: the proteomics server for in-depth protein knowledge and analysis. Nucleic Acids Res. (2003) 31:3784–8. doi: 10.1093/nar/gkg563 12824418 PMC168970

[24] Laskowski RA . PDBsum1: A standalone program for generating PDBsum analyses. Protein Sci. (2022) 31:e4473. doi: 10.1002/pro.4473 36251626 PMC9667822

[25] Zhou X Zheng W Li Y Pearce R Zhang C Bell EW . I-TASSER-MTD: a deep-learning-based platform for multi-domain protein structure and function prediction. Nat Protoc. (2022) 17:2326–53. doi: 10.1038/s41596-022-00728-0 35931779

[26] Heo L Park H Seok C . GalaxyRefine: Protein structure refinement driven by side-chain repacking. Nucleic Acids Res. (2013) 41:W384–8. doi: 10.1093/nar/gkt458 23737448 PMC3692086

[27] Wiederstein M Sippl MJ . ProSA-web: interactive web service for the recognition of errors in three-dimensional structures of proteins. Nucleic Acids Res. (2007) 35:W407–10. doi: 10.1093/nar/gkm290 17517781 PMC1933241

[28] Mendes M Mahita J Blazeska N Greenbaum J Ha B Wheeler K . IEDB-3D 2.0: structural data analysis within the Immune Epitope Database. Protein Sci. (2023) 32:e4605. doi: 10.1002/pro.4605 36806329 PMC10022491

[29] Vita R Blazeska N Marrama D IEDB Curation Team Members Duesing S Bennett J . The immune epitope Database (IEDB): 2024 update [Database. Nucleic Acids Res. (2025) 53:D436–43. doi: 10.1093/nar/gkae1092 39558162 PMC11701597

[30] Craig DB Dombkowski AA . Disulfide by Design 2.0: a web-based tool for disulfide engineering in proteins. BMC Bioinform. (2013) 14:346. doi: 10.1186/1471-2105-14-346 24289175 PMC3898251

[31] Rošinec A Slanináková T Pavlík T Randiak R Svoboda T Raček T . Gromacs MetaDump: a tool for extracting GROMACS simulation metadata. J Cheminform. (2025) 17:160. doi: 10.1186/s13321-025-01082-5 41131648 PMC12548288

[32] Maier JA Martinez C Kasavajhala K Wickstrom L Hauser KE Simmerling C . ff14SB: improving the accuracy of protein side chain and backbone parameters from ff99SB. J Chem Theory Comput. (2015) 11:3696–713. doi: 10.1021/acs.jctc.5b00255 26574453 PMC4821407

[33] Rosas Jiménez JG Fábián B Hummer G . Faster sampling in Molecular Dynamics simulations with TIP3P-F water. J Chem Theory Comput. (2024) 20:11068–81. doi: 10.1021/acs.jctc.4c00990 39668361 PMC11672673

[34] Duan Q Flynn C Niepel M Hafner M Muhlich JL Fernandez NF . Lincs Canvas Browser: interactive web app to query, browse and interrogate Lincs L1000 gene expression signatures. Nucleic Acids Res. (2014) 42:W449–60. doi: 10.1093/nar/gku476 24906883 PMC4086130

[35] Rapin N Lund O Bernaschi M Castiglione F . Computational immunology meets bioinformatics: the use of prediction tools for molecular binding in the simulation of the immune system. PloS One. (2010) 5:e9862. doi: 10.1371/journal.pone.0009862 20419125 PMC2855701

[36] Moxon R Reche PA Rappuoli R . Editorial: reverse vaccinology. Front Immunol. (2019) 10:2776. doi: 10.3389/fimmu.2019.02776 31849959 PMC6901788

[37] Yin Z Li M Niu C Yu M Xie X Haimiti G . Design of multi-epitope vaccine candidate against Brucella type IV secretion system (T4SS). PloS One. (2023) 18:e0286358. doi: 10.1371/journal.pone.0286358 37561685 PMC10414599

[38] Sethi G Sethi S Krishna R . Multi-epitope based vaccine design against Staphylococcus epidermidis: A subtractive proteomics and immunoinformatics approach. Microb Pathog. (2022) 165:105484. doi: 10.1016/j.micpath.2022.105484 35301068

[39] Swetha RG Basu S Ramaiah S Anbarasu A . Multi-epitope vaccine for monkeypox using pan-genome and reverse vaccinology approaches. Viruses. (2022) 14:2504. doi: 10.3390/v14112504 36423113 PMC9695528

[40] Zhu L Cui X Yan Z Tao Y Shi L Zhang X . Design and evaluation of a multi-epitope DNA vaccine against HPV16. Hum Vaccin Immunother. (2024) 20:2352908. doi: 10.1080/21645515.2024.2352908 38780076 PMC11123455

[41] Ahmed N Rani NA Robin TB Mashrur MN Shovo MMI Prome AA . Designing a multi-epitope subunit vaccine against Toxoplasma gondii through reverse vaccinology approach. Mol Biochem Parasitol. (2024) 260:111655. doi: 10.1016/j.molbiopara.2024.111655 39521441

[42] Newman MJ Livingston B McKinney DM Chesnut RW Sette A . T-lymphocyte epitope identification and their use in vaccine development for HIV-1. Front Biosci. (2002) 7:d1503–15. doi: 10.2741/A730 12048179

[43] Leung K . (99m)Tc-Human β-defensin-3. In: Molecular Imaging and Contrast Agent Database (MICAD) Bethesda (MD): National Center for Biotechnology Information (US). (2009). 20641179

[44] Devarakonda Y Reddy MVNJ Neethu RS Chandran A Syal K . Multi epitope vaccine candidate design against Streptococcus pneumonia. J Biomol Struct Dyn. (2023) 41:12654–67. doi: 10.1080/07391102.2023.2167123 36636838

[45] Zhu X Wang X Liu T Zhang D Jin T . Design of multi-epitope vaccine against porcine rotavirus using computational biology and molecular dynamics simulation approaches. Virol J. (2024) 21:160. doi: 10.1186/s12985-024-02440-9 39039549 PMC11264426

